# Tiagabine and zonisamide differentially regulate the glial properties in an astrocyte-microglia co-culture model of inflammation

**DOI:** 10.1007/s00210-023-02538-x

**Published:** 2023-05-25

**Authors:** Fatme Seval Ismail, Pedro M. Faustmann, Eckart Förster, Franco Corvace, Timo Jendrik Faustmann

**Affiliations:** 1 Department of Neurology, University Hospital Knappschaftskrankenhaus Bochum, Ruhr University Bochum, Bochum, Germany; 2 Department of Neuroanatomy and Molecular Brain Research, Medical Faculty, Ruhr University Bochum, Bochum, Germany; 3 Department of Psychiatry and Psychotherapy, Medical Faculty, Heinrich Heine University, Düsseldorf, Germany

**Keywords:** Tiagabine, Zonisamide, Astrocyte-microglia co-culture model of inflammation, Connexin 43, Gap junctions, Toxicity

## Abstract

Due to the role of astrocytes and microglia in the pathophysiology of epilepsy and limited studies of antiseizure medication (ASM) effects on glial cells, we studied tiagabine (TGB) and zonisamide (ZNS) in an astrocyte-microglia co-culture model of inflammation. Different concentrations of ZNS (10, 20, 40, 100 µg/ml) or TGB (1, 10, 20, 50 µg/ml) were added to primary rat astrocytes co-cultures with 5–10% (M5, physiological conditions) or 30–40% (M30, pathological inflammatory conditions) microglia for 24 h, aiming to study glial viability, microglial activation, connexin 43 (Cx43) expression and gap-junctional coupling. ZNS led to the reduction of glial viability by only 100 µg/ml under physiological conditions. By contrast, TGB revealed toxic effects with a significant, concentration-dependent reduction of glial viability under physiological and pathological conditions. After the incubation of M30 co-cultures with 20 µg/ml TGB, the microglial activation was significantly decreased and resting microglia slightly increased, suggesting possible anti-inflammatory features of TGB under inflammatory conditions. Otherwise, ZNS caused no significant changes of microglial phenotypes. The gap-junctional coupling was significantly decreased after the incubation of M5 co-cultures with 20 and 50 µg/ml TGB, which can be related to its anti-epileptic activity under noninflammatory conditions. A significant decrease of Cx43 expression and cell–cell coupling was found after the incubation of M30 co-cultures with 10 µg/ml ZNS, suggesting additional anti-seizure effects of ZNS with the disruption of glial gap-junctional communication under inflammatory conditions. TGB and ZNS differentially regulated the glial properties. Developing novel ASMs targeting glial cells may have future potential as an “add-on” therapy to classical ASMs targeting neurons.

## Introduction

Epilepsy is associated with the predisposition to generate recurrent epileptic seizures due to abnormal excessive or synchronous neuronal excitation in the brain, resulting in neurobiological, cognitive, psychological and social consequences (Fisher et al. 2014; McCormick and Contreras 2001; Thijs et al. 2019). More than 50 to 70 million people worldwide are affected by epilepsy (Fiest et al. 2017; Ngugi et al. 2011; Thijs et al. 2019). Treatment with antiseizure medications (ASMs) affecting neuronal cells is the main therapeutic approach (Perucca and Tomson 2011; Rogawski et al. 2016; Thijs et al. 2019). However, therapy refractory epilepsy occurs in about 30% of patients (Löscher et al. 2020). Following this, treatment of seizures with ASMs can be difficult depending on various mechanisms, and further understanding of pathophysiological mechanisms in epilepsy and drug resistance is required (Löscher et al. 2020; Perucca et al. 2007). Glia and inflammation can contribute to seizure generation in many ways (Devinsky et al. 2013; Eyo et al. 2017; Patel et al. 2019). On the one hand, epileptic seizures lead to the activation of different inflammatory cascades, including the activation of astrocytes and microglia (Vezzani et al. 2011; Vezzani and Granata 2005). On the other hand, autoimmune inflammation, for example, antibody-associated autoimmune encephalitis, leads to epileptic seizures (Dalmau and Graus 2018; Geis et al. 2019). Astrocytes, the main glia cell population in the central nervous system, support brain homeostasis, stabilize neuronal networks and form part of the blood–brain barrier in the healthy (Dermietzel et al. 1991; Giaume et al. 1991, 2010; Siracusa et al. 2019). Astrocytic networks are connected by the gap junctional protein connexin 43 (Cx43), which can contribute to epileptogenesis and further support the role of glial cells in the generation of seizures (Giaume et al. 2010; Mylvaganam et al. 2010, 2014). Gap junctions (GJs) contribute to direct cell-to-cell communication and intercellular exchange (Dermietzel et al. 1991; Giaume et al. 1991, 2010). In addition to enhanced gap junctional communication between neurons, astrocytic gap junctional communication was also related to seizure activity (Mylvaganam et al. 2014). Microglia react to pathological conditions with proliferation and cytokine release, for example, the morphological and molecular activation of microglia in epilepsy has been observed (Eyo et al. 2017). Amounts of microglia can increase from 5–20% of the glial cell population in the healthy brain up to 30% or more under inflammatory conditions, accompanied by morphological changes from a resting ramified (RRT) to a rounded phagocytic type (RPT) (Faustmann et al. 2003). Previous studies showed that microglia may influence neuronal activity in epilepsy and astrocyte-mediated excitation (Eyo et al. 2017; Pascual et al. 2012; Patel et al. 2019; Vezzani et al. 2011, 2013). The role of inflammation in epilepsy is strongly discussed and also underlines an important role of microglia in this context (Aronica et al. 2017; Pascual et al. 2012; Vezzani et al. 2011, 2013; Vezzani and Granata 2005). An important step in this direction is the development of an astrocyte-microglia co-culture model by Faustmann et al. (2003), which is suitable for studying pharmacological effects under physiological and pathological inflammatory conditions involving glial cells (Faustmann et al. 2003; Ismail et al. 2021a). Effects of ASMs, such as lacosamide, lamotrigine, topiramate, levetiracetam, brivaracetam, valproic acid, gabapentin, phenytoin and carbamazepine, on glial cells have been investigated to date in terms of inflammation, mimicking conditions under severe diseases of the central nervous system, such as epilepsy (Corvace et al. 2022; Dambach et al. 2014; Faustmann et al. 2022; Haghikia et al. 2008; Ismail et al. 2022; Ismail et al. 2021a; Ismail and Faustmann 2021; Stienen et al. 2011).

Tiagabine (TGB) (R-N-(4,4-di(3-methylthien-2-yl)-but-3-enyl-nipecotic acid hydrochloride) is a new-generation ASM approved by the European Medicines Agency and the United States Food and Drug Administration as adjunctive therapy for use in adults and children 12 years and older with refractory partial seizures. Tiagabine is a potent inhibitor of presynaptic γ-aminobutyric acid uptake in neuronal and glial cells due to the inhibition of the γ-aminobutyric acid transporter 1 (GAT-1) (Ängehagen et al. 2003; Brodie 1995; Fraser et al. 1999a; Leach et al. 1996; Suzdak and Jansen 1995). As has been shown previously, TGB at the usual recommended doses did not induce DNA fragmentation in cortical rat astrocytes or exert toxic effects, but DNA damage was observed at very high concentrations (Cardile et al. 2000, 2001). In addition, protective effects of TGB against oxidative stress on astrocytes were described (Cardile et al. 2000). Moreover, TGB attenuated microglial activation and protected dopaminergic neurons against neurotoxins in this way (Liu et al. 2015).

Zonisamide (ZNS) (1,2-benzisoxazole-3-methaesulfonamide) is a new-generation ASM approved by the European Medicines Agency and the Food and Drug Administration for the monotherapy of partial seizures in adults and adjunctive therapy of partial seizures in adults and children older than 6 years. Mechanisms of action of ZNS include alteration of the fast inactivation threshold of voltage-dependent sodium channels, leading to a reduction of high-frequency repetitive firing of action potentials, inhibition of low-threshold T-type calcium channels in neurons, a weak inhibition of carbonic anhydrase and additional effects on dopamine, serotonin and acetylcholine metabolism (Biton 2007; Walker and Patsalos 1995). It has been shown that ZNS increased glutathione in astrocytes, leading to neuroprotection against oxidative stress (Asanuma et al. 2010). In addition, increased expression of astrocyte-mediated neurotrophic and anti-oxidative factors induced by ZNS was related to neuroprotection (Choudhury et al. 2012). The ZNS showed effects on gliotransmitter release via the modulation of astroglial hemichannel function, such as decreased Cx43 expression and inhibited Cx43 hemichannel activity (Fukuyama et al. 2020). Moreover, ZNS decreased Iba1-positive microglia in the spinal cord in a mouse model of neuropathic pain and reduced amoeboid-shaped activated microglia in BV2 microglial cell lines, suggesting effects against neuropathic pain by the inhibition of microglial activation (Koshimizu et al. 2020).

The increasing evidence confirming the role of astrocytes and microglia in the pathophysiology of epilepsy and limited studies of ASM effects on glial cells underline TGB and ZNS as interesting targets to study in our astrocyte-microglia co-culture model. Therefore, we aimed to investigate effects of TGB and ZNS on glial viability, microglial phenotypes, expression of GJ protein Cx43 and intercellular communication under physiological and pathological inflammatory conditions of the astrocyte-microglia co-culture model of inflammation.

## Material and methods

### Cell culture

Faustmann et al. (2003) developed a protocol for the preparation of primary astrocyte-microglia co-cultures. The so-called “M5” astrocyte-microglia co-cultures are consistent with physiological conditions in the brain and contain an average of 5–10% microglia with a predominantly resting, inactivated phenotype, whereas the “M30” co-cultures contain an average of 30–40% microglia with a predominantly activated phenotype and correspond to pathological, inflammatory conditions. Previously, the pro-inflammatory cytokine tumor necrosis factor (TNF)-α in our astrocyte-microglia co-culture model was increased after 2 and 24 h of stimulation with gram-negative-derived lipopolysaccharide together with an increase of the activated phenotype of microglia from 24.69% in the control to 40.54% after 2 h and 72% after 24 h, confirming that the amount of increased and activated microglia correlates with the concentration of TNF-α. Interestingly, this effect could be reversed by a co-incubation with anti-inflammatory dexamethasone, demonstrating the proof of principle of our inflammatory M30 co-culture (Hinkerohe et al. 2010). In summary, these findings confirm the M30 model as an inflammatory condition. The co-culture model was also used previously in other pharmacological studies (Corvace et al. 2022, 2023; Dambach et al. 2014; Faustmann et al. 2022; Ismail et al. 2022; Ismail et al. 2021a). According to current animal protection regulations in Germany (Animal Welfare Act) and within the European Union, the removal of organs or cells from vertebrates for scientific purposes is not considered an animal experiment if the animals have not been subject to surgical interventions or invasive treatments prior to sacrifice. Thus, euthanization of postnatal rats intended for the removal of brain tissue in this study does not need approval or permission from local or governmental authorities. An ethics approval is deemed unnecessary according to the relevant German (Animal Welfare Act) and European (Directive 2010/63/EU) legislation. All experimental protocols used in the research project to generate primary cell cultures were carried out in compliance with the regulations of the Animal Welfare Act and animal protection regulations applicable within the European Union.

The cerebral hemispheres were obtained from postnatal Wistar rats (P0-P2) after decapitation without sedation in compliance with the German Animal Protection Act. After removal of the meninges and the choroid plexus, collection of brain tissue in ice-cold phosphate buffered saline (PBS) (preparation of 1X from 10X stock solution, consisting of 1.38 M NaCl, 27 mM KCl, 81 mM Na_2_HPO_4_, 14.7 mM KH_2_PO_4_) (J.T. Baker, Deventer, the Netherlands) and then treatment with 0.1% trypsin solution (30 min at 37 °C) was performed. The next steps included: **1)** centrifugation at 500 × g for 12 min at room temperature; **2)** resuspension of the pellet in 5 ml of DNase I solution (Serva Electrophoresis, Heidelberg, Germany) (100 µl/ml with Dulbecco’s minimal essential medium: DMEM, Invitrogen, Karlsruhe, Germany) and incubation for 5 min at room temperature; **3)** filling up with wash medium, consisting of DMEM, 10% fetal calf serum (Biochrom AG, Berlin, Germany), 1% penicillin/streptomycin solution (PAA Laboratories, Linz, Austria), to a total volume of 20 ml and re-centrifugation (5 min at 200 × g); **4)** resuspension of pellet in 2 ml DMEM per brain and filtration through a nylon filter with a pore size of 60 µm; **5)** seeding in cell culture flasks (2–4 brains per flask) and incubation at 5% CO_2_ and 37 °C in astrocyte culture medium, consisting of DMEM, 10% fetal calf serum, 1% nonessential amino acids, 1% glutamine, 1% penicillin/streptomycin solution (PAA Laboratories, Linz, Austria); **6)** washing of adherent cells the next day and adding a new cell medium; **7)** passage and preparation of the cells for further experiments after reaching 100% confluence on about days 5–7, and finally, **8)** both M5 and M30 co-culture conditions could be distinguished based on the microglia percentage, which was determined depending on the extent of shaking.

### Treatment of cultures

Considering previous study findings with ZNS and TGB, both M5 and M30 co-cultures were incubated with different concentrations of either ZNS (10, 20, 40 or 100 µg/ml) (Sigma-Aldrich, Taufkirchen, Germany) or TGB (1, 10, 20 or 50 µg/ml) (Sigma-Aldrich, Schnelldorf, Germany) (Adkins and Noble 1998; Asanuma et al. 2010; Biton 2007; Cardile et al. 2000, 2001; Fraser et al. 1999b; Masuda et al. 1979; Pavone and Cardile 2003; Perucca 1999; Perucca and Bialer 1996; Walker and Patsalos 1995; Wang et al. 2004). The appropriate concentration (drugs dissolved in distilled H_2_O according to the manufacturer’s instructions) for incubation was added directly to the medium of each cell culture and incubated for 24 h in 5% CO_2_ at 37 °C. The high concentrations (100 µg/ml ZNS; 20 and 50 µg/ml TGB) were used to reveal toxic effects (Cardile et al. 2001; Masuda et al. 1979). Co-cultures untreated with any substance/vehicle were called control 1 and those incubated with the vehicle distilled H_2_O (5 or 20 µl per ml cell culture medium) were called control 2.

### MTT assay

An MTT assay (3-(4,5-dimethylthiazol-2-yl)-2,5-diphenyltetrazolium bromide) (Roche applied sciences, Penzberg, Germany) was performed to study the TGB and ZNS effects in terms of proliferation, toxicity and viability on our co-cultures. Cells were seeded at a density of 20,000 cells per well on a 96-well plate and incubated at confluence with TGB and ZNS at the concentrations mentioned previously. According to the protocol, firstly, 10 μl MTT reagent was added to each well for 4 h at 37 °C and 5% CO_2_, and a subsequent incubation with 100 µl of solubilization solution per well was performed overnight (Corvace et al. 2022, 2023; Faustmann et al. 2022; Ismail et al. 2022). The next day, the Bio-Rad microplate reader (München, Germany) was used to measure the absorbance at a wavelength of 550 nm.

### Immunocytochemistry

**Fig. 1:**
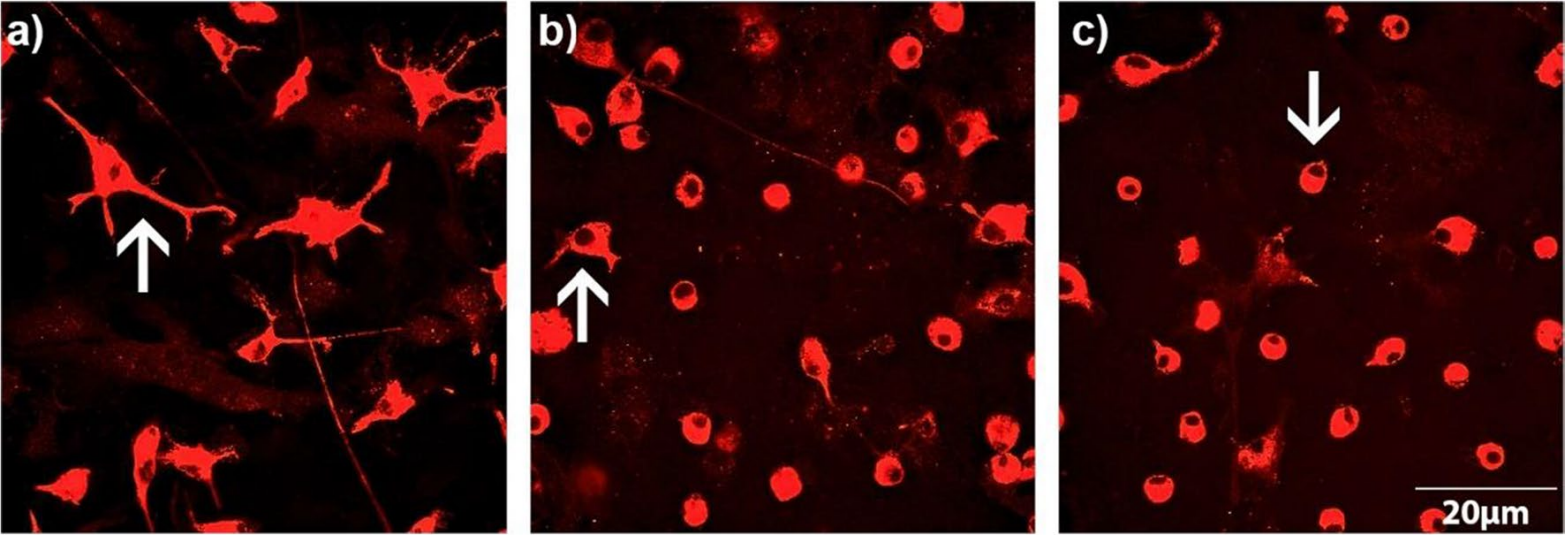
Microglial morphology by immunocytochemistry in an astrocyte-microglia co-culture model of inflammation after treatment with tiagabine (TGB) and zonisamide (ZNS). The monoclonal antibody ED1 (red) led to the classification of all microglial phenotypes as resting ramified (RRT), intermediate and activated rounded phagocytic (RPT) phenotypes (*white arrows*) at a magnification of 600 × (**a-c**). The RRT microglial phenotype is characterized by small cell bodies with a small cytoplasmic rim and thin branching processes longer than the diameter of the cell body (**a**). Typical features of the intermediate phenotype are a few vesicles and vacuoles in the cytoplasmic rim with some thick pseudopodia longer than the diameter of the cell body (**b**). The activated RPT microglial phenotype is characterized by a large cellular diameter with several cytoplasmic vacuoles and rare short processes (**c**). A bar indicates 20 µm

Immunocytochemistry was performed to study the effects of both drugs on microglial phenotypes and Cx43 signaling. The ED1 antibody acted as the strongest microglial marker in experiments performed by Faustmann et al. (2003). The ED1 staining led to the classification of all microglial phenotypes as resting RRT, intermediate and activated RPT phenotypes (Fig. 1 a-c). Typical features of RRT microglial phenotype are small cell bodies with a small cytoplasmic rim and thin branching processes longer than the diameter of the cell body (Fig. 1 a). The intermediate phenotype is characterized by a few vesicles and vacuoles in the cytoplasmic rim with some thick pseudopodia longer than the diameter of the cell body (Fig. 1 b). The activated RPT microglial phenotype is distinguished by a large cellular diameter with several cytoplasmic vacuoles and rare short processes (Fig. 1 c). The immunocytochemical protocol of this study was used in previous pharmacological studies (Corvace et al. 2022, 2023; Faustmann et al. 2022; Ismail et al. 2022). In preparation for immunocytochemistry, the 24 well-plates were filled with cover slips coated with poly-L-lysine. Cells were then seeded at a density of 70,000 cells per well and incubated at 37 °C and 5% CO_2_. Upon reaching at least 80% confluence, cells were incubated with the concentrations of TGB or ZNS mentioned previously for 24 h. Following this, cover slips were washed with PBS and fixed with ice-cooled ethanol (100%) for 10 min. The cells were washed again three times with PBS and blocked with PBS-blocking solution, consisting of 1% bovine serum albumin and 10% horse serum (PAA Laboratories, Linz, Austria), for 1 h. The primary antibody (mouse) anti-ED1 (anti-CD68) (1:250) (Bio-Rad Laboratories, Feldkirchen, Germany) and the primary antibody (rabbit) anti-Cx43 (1:1000) (Invitrogen, Karlsruhe, Germany) were prepared in PBS-blocking solution (PAA Laboratories, Linz, Austria). Following this, 25 µl of antibody solution were added per coverslip and incubated overnight at 4 °C. After washing the wells three times with PBS and 1% bovine serum albumin, the secondary antibodies Alexa fluor® 568 (mouse) (1:500) and Alexa fluor® 488 (rabbit) (1:500) (Invitrogen, Karlsruhe, Germany) dissolved in PBS-blocking solution were also added to the cover slips (25 µl per well) and incubated for 1 h. The well plate was then washed three times with PBS for 10 min each time. In the last step, the cover slips were incubated with the nuclear dye DAPI (4′,6-diamidine-2-phenylindole) (1:1000) (Invitrogen, Karlsruhe, Germany) for the quantification of the cell numbers. ED1-stained microglia were compared with the total number of DAPI-labeled cells to assess the microglia-astrocytes ratio. The microglia phenotypes and Cx43 expression were evaluated in a minimum of three different visual fields on each cover slip at a primary magnification of 600 × using a Zeiss Axiovert 100 M laser scanning confocal microscope (Carl Zeiss, Jena, Germany). The ImageJ software program (Rasband, W.S. ImageJ. National Institutes of Health, Bethesda, Maryland, USA) allowed us to analyze the immunocytochemical Cx43 signal per cell.

### Scrape loading and dye transfer

The method of scrape loading dye transfer was performed to study the gap-junctional coupling and the effect of drugs on it (El-Fouly et al. 1987). Cells were incubated on cover slips at a density of 70,000 cells per well, similar to the immunocytochemistry described, and incubated with the drugs and concentrations mentioned previously for 24 h when at least 80% confluency was reached. The next steps included: **1)** washing with PBS for 2 × 1 min and adding 400 μl of 0.05% (w/v) Lucifer Yellow CH solution (in PBS) (Thermo Fisher Scientific, Dreieich, Germany) per well; **2)** an incision on the confluent cell surface as straight as possible from left to right with an injection cannula (0.45 × 12 mm); **3)** incubation with Lucifer Yellow CH solution for 2 min (37 °C) under exclusion of light; **4)** washing again with PBS; and **5)** a direct evaluation using the Zeiss Axiovert 100 M laser scanning confocal microscope (Carl Zeiss, Jena, Germany) at 10 × magnification (wavelength of 488 nm). The distribution of Lucifer Yellow (LY) in the entire area was reported in % using the ImageJ software program (Rasband, W.S. ImageJ. National Institutes of Health, Bethesda, Maryland, USA).

### Data analyses and statistics

Microsoft Excel 365 and GraphPad Prism 7.0 for Windows (GraphPad Software, San Diego, USA) were performed for statistical analyses. D’Agostino-Pearson omnibus tests were performed to analyze the normality of the data distribution. Parametric tests were performed in the case of normality. Comparisons between more than two groups with normal distribution were evaluated using one-way analysis of variance (one-way ANOVA) followed by a Kruskal–Wallis test or Bonferroni post hoc comparison test. The significance was defined as p < 0.05. The results were shown as mean ± standard error of the mean.

## Results

### Effects of TGB and ZNS on glial cell viability

**Fig. 2:**
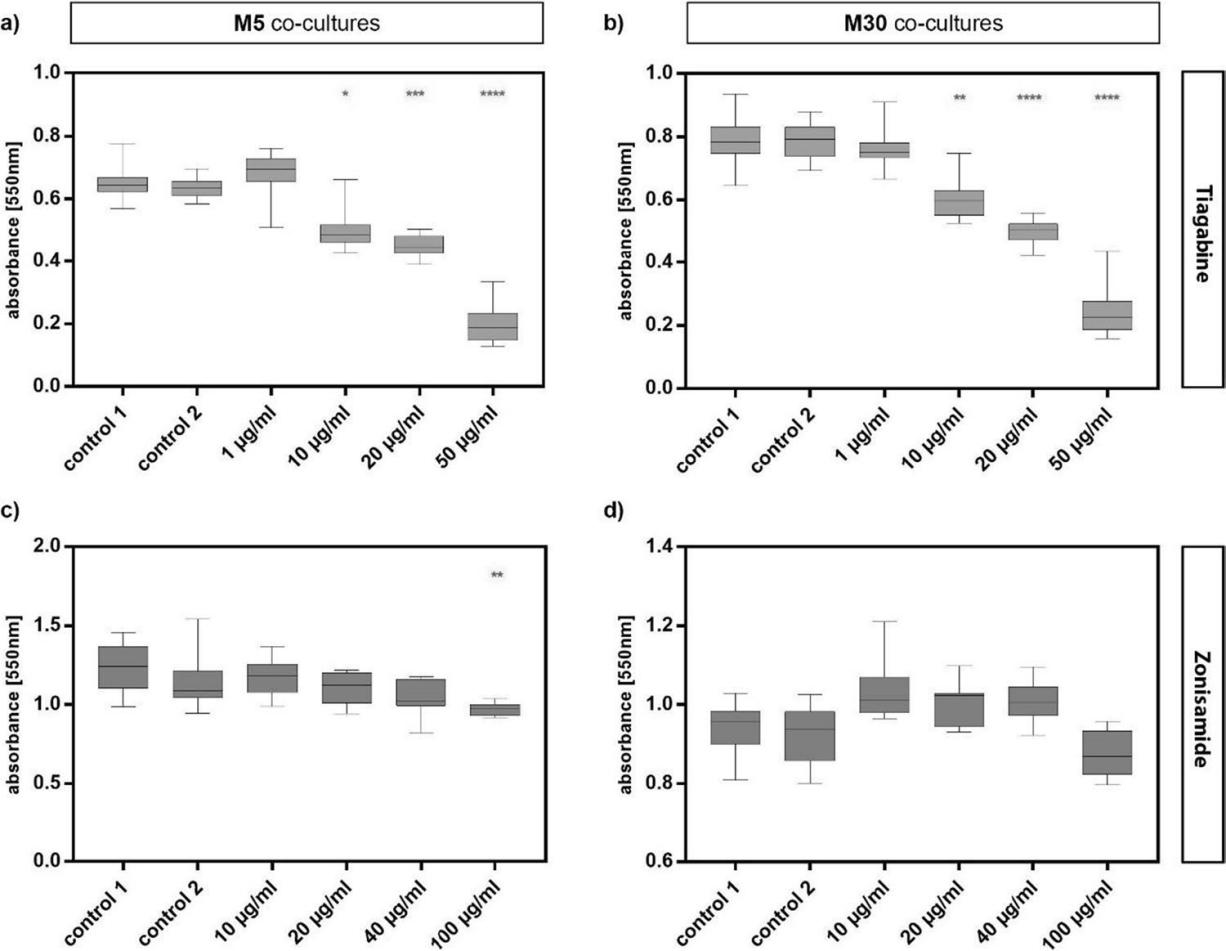
Glial viability detected by MTT assay in physiological M5 (**a, c**) and pathological M30 (**b, d**) astrocyte-microglia co-cultures after concentration-dependent incubation with either zonisamide (ZNS) (10, 20, 40 or 100 µg/ml) or tiagabine (TGB) (1, 10, 20 or 50 µg/ml) for 24 h. The glial viability was significantly decreased after incubation with 10, 20 and 50 µg/ml TGB of M5 (10 µg/ml p < 0.05: *; 20 µg/ml p < 0.001: ***; 50 µg/ml p < 0.0001: ****) (n = 16) (**a**) and M30 (n = 16) (10 µg/ml p < 0.01: **; 20 and 50 µg/ml p < 0.0001: ****) (**b**) co-cultures compared to both controls. Higher concentrations of TGB in M5 co-cultures caused a significant reduction of glial viability compared to 1 µg/ml TGB (10 µg/ml p < 0.001: ***; 20 and 50 µg/ml p < 0.0001: ****). Amounts of 20 (p < 0.001) and 50 (p < 0.0001: ****) µg/ml TGB in M30 co-cultures also significantly reduced the glial cell viability compared to 1 µg/ml TGB. The glial cell viability was significantly decreased after incubation with 100 µg/ml ZNS of M5 (n = 8) co-cultures compared to both controls (p < 0.001: ***) (**c**) and to 10 µg/ml (p < 0.05: *). Different concentrations of ZNS in pathological M30 co-cultures caused no significant changes (n = 8) (**d**). Comparisons between the groups were performed using the D’Agostino-Pearson normality test and one-way analysis of variance (one-way ANOVA) followed by a Kruskal–Wallis test. Differences were considered significant at p < 0.05: *, p < 0.01: **, p < 0.001: ***, p < 0.0001: ****. Control 1: untreated cells with any substance/vehicle; Control 2: cells incubated with the vehicle distilled H_2_O (5 or 20 µl per ml cell culture medium)

Co-cultures (physiological M5 and pathological M30) were incubated with 1, 10, 20 and 50 µg/ml of TGB and 10, 20, 40 and 100 µg/ml of ZNS for 24 h. A significant concentration-dependent decrease of glial cell viability was detected after incubation with 10, 20 and 50 µg/ml TGB of M5 (Fig. 2 a) and M30 (Fig. 2 b) co-cultures compared to both controls (untreated cells as control 1 and cells incubated with distilled H20 as control 2). Compared to incubation with 1 µg/ml TGB, treatment with higher concentrations of TGB (10, 20 and 50 µg/ml) led to a significant reduction of glial viability in M5 co-cultures. Incubation of M30 co-cultures with 20 and 50 µg/ml TGB reduced the glial cell viability significantly compared to incubation with 1 µg/ml TGB. After incubation of M5 co-cultures with 100 µg/ml ZNS, the glial cell viability was significantly decreased compared to both controls (Fig. 2 c) and incubation with 10 µg/ml. However, the incubation of pathological M30 co-cultures with different concentrations of ZNS revealed no significant changes (Fig. 2 d).

### Effects of TGB and ZNS on microglial activation under physiological and pathological conditions

**Fig. 3:**
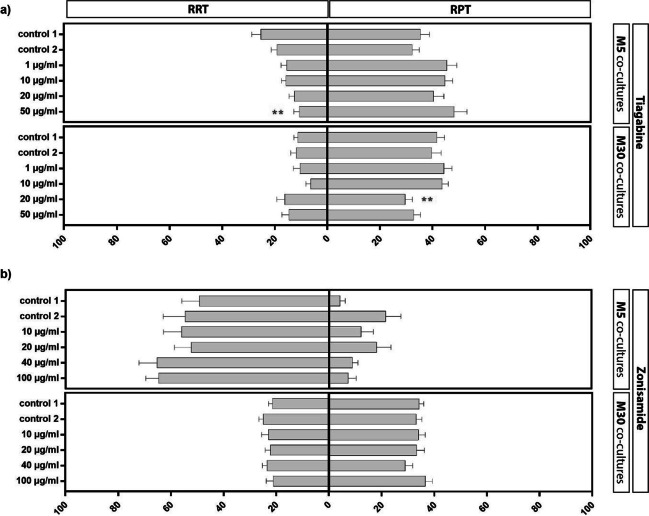
Microglial phenotypes identified by immunocytochemistry as resting ramified type (RRT) and rounded phagocytic type (RPT) in physiological M5 and pathological M30 co-cultures after concentration-dependent incubation with either zonisamide (ZNS) (10, 20, 40 or 100 µg/ml) (**b**) or tiagabine (TGB) (1, 10, 20 or 50 µg/ml) (**a**) for 24 h. The incubation with 50 µg/ml of TGB in M5 co-culture significantly reduced the amount of resting RRT microglia compared to both controls (p < 0.01: **) (n = 29) (**a**). The incubation with 20 µg/ml TGB in M30 co-cultures significantly reduced the amount of activated RPT microglia (F = 4.43; p < 0.01: **) (n = 26) (**a**). The incubation of M5 (n = 9) and M30 (n = 30) co-cultures with different concentrations of ZNS (F = 0.4288) caused no significant changes compared to both controls (**b**). Comparisons between the groups were performed using the D’Agostino-Pearson normality test and one-way ANOVA followed by a Bonferroni post hoc comparison test or Kruskal–Wallis test. Differences were considered significant at p < 0.01: **. Control 1: untreated cells with any substance/vehicle; Control 2: cells incubated with the vehicle distilled H_2_O (5 or 20 µl per ml cell culture medium)

After adding 50 µg/ml of TGB to physiological M5 co-cultures for 24 h, the amount of the resting RRT of microglia was significantly decreased compared to both controls (untreated cells as control 1 and cells incubated with distilled H_2_O as control 2) with a parallel slight, but not significant, increased activated microglia (Fig. 3 a). After incubation of pathological M30 co-cultures with 20 µg/ml TGB, the amount of the activated RPT of microglia was significantly decreased and, additionally, the amount of resting microglia was slightly, but not significantly, increased (Fig. 3 a). Under all other conditions, the microglial phenotypes were not affected by TGB.

Incubation of physiological M5 and pathological M30 co-cultures with different concentrations of ZNS resulted in no significant changes compared to both controls (Fig. 3 b).

The total amount of glial and microglial cells was not significantly changed after incubation with different concentrations of TGB or ZNS (data not shown).

### Modulation of Cx43 expression after incubation with TGB and ZNS in physiological and pathological astrocyte-microglia co-cultures

**Fig. 4:**
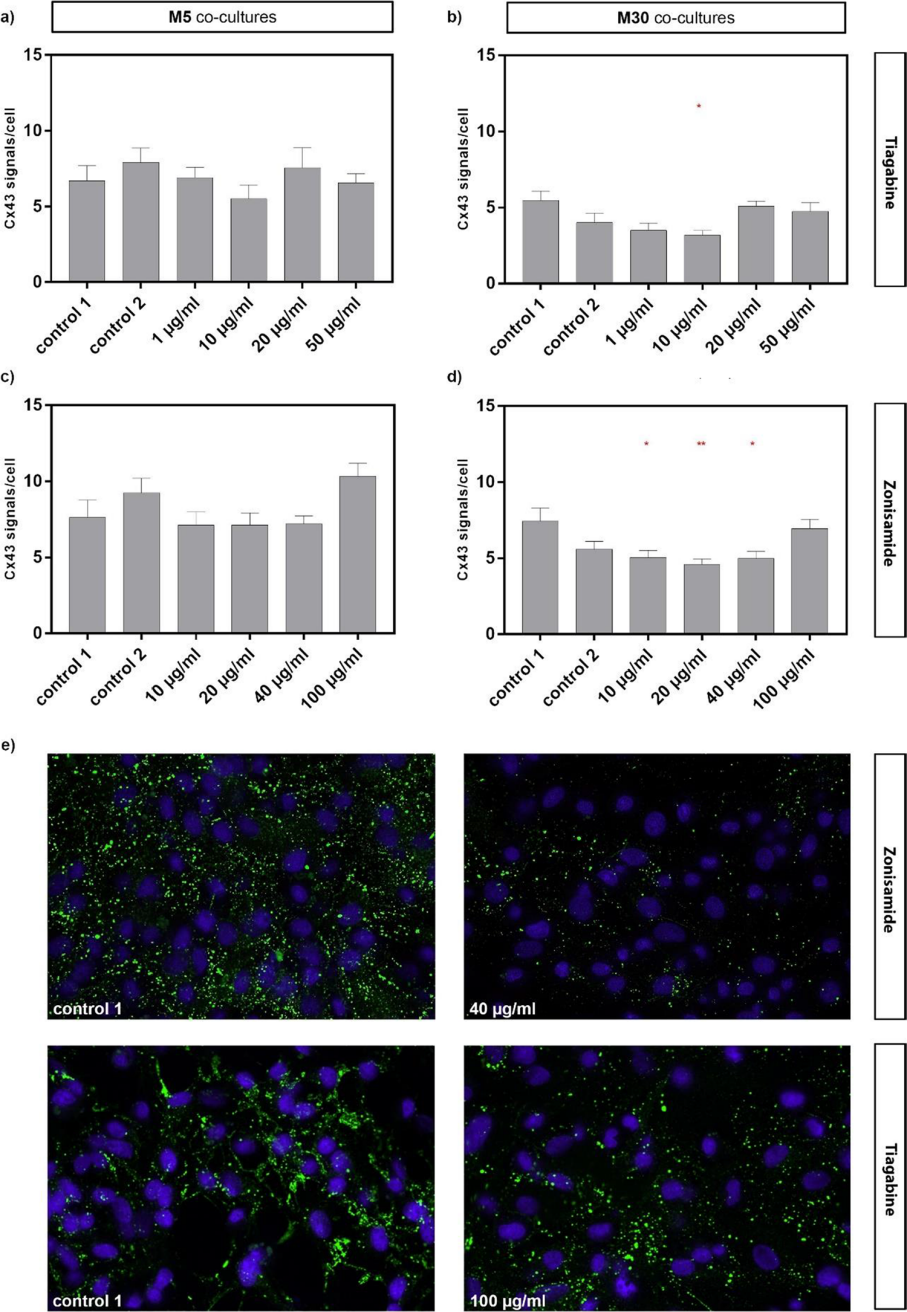
Immunocytochemical staining of connexin 43 (Cx43) after the concentration-dependent incubation with either zonisamide (ZNS) (10, 20, 40 or 100 µg/ml) (**c, d**) or tiagabine (TGB) (1, 10, 20 or 50 µg/ml) (**a, b**) for 24 h in physiological M5 and pathological M30 astrocyte-microglia co-cultures. The Cx43 expression was not affected after the incubation with 1, 10, 20 and 50 µg/ml TGB for 24 h in M5 co-cultures (detection of Cx43 signal/cell) (n = 10), but a significant (p < 0.05: *) (n = 12) reduction was detected after the incubation with 10 µg/ml TGB in M30 co-cultures compared to both controls (**a, b**). The incubation of M5 co-cultures with 10, 20, 40 and 100 µg/ml ZNS caused no significant changes in the Cx43 signal/cell (F = 2.496; n = 9). However, the Cx43 was significantly reduced after incubation with 10 µg/ml (F = 4.187; p < 0.05: *) (n = 29), 20 µg/ml (p < 0.01: **) (n = 31) and 40 µg/ml (p < 0.05: *) (n = 26) ZNS in M30 co-cultures compared to both controls (**c, d**). The panel **e** shows an example of Cx43 staining of control 1 compared to the incubation with 40 μg/ml ZNS and control 1 compared to the incubation with 100 μg/ml TGB of the pathological M30 co-culture (cell nuclei stained with DAPI: blue; Cx43 signal: green). Comparisons between the groups were performed using the D’Agostino-Pearson normality test and one-way ANOVA followed by a Bonferroni post hoc comparison test or Kruskal–Wallis test. Differences were considered significant at p < 0.05: *, p < 0.01: **. Control 1: untreated cells with any substance/vehicle; Control 2: cells incubated with the vehicle distilled H_2_O (5 or 20 µl per ml cell culture medium)

The detection of Cx43 expression by immunocytochemistry resulted in no significant changes after the incubation with 1, 10, 20 and 50 µg/ml TGB for 24 h in physiological M5 co-cultures (detection of Cx43 signal/cell), but in a significant reduction after the incubation with 10 µg/ml TGB in pathological M30 co-cultures compared to both controls (untreated cells as control 1 and cells incubated with distilled H_2_O as control 2) (Fig. 4 a, b).

No significant changes were detected after the incubation of physiological M5 co-cultures with 10, 20, 40 and 100 µg/ml ZNS for 24 h (detection of Cx43 signal/cell), but a significant reduction of Cx43 expression was found after the incubation of M30 co-cultures with 10, 20 and 40 µg/ml ZNS compared to both controls (untreated cells as control 1 and cells incubated with distilled H_2_O as control 2) (Fig. 4 c, d).

### Effects of TGB and ZNS on glial cell–cell communication

**Fig. 5:**
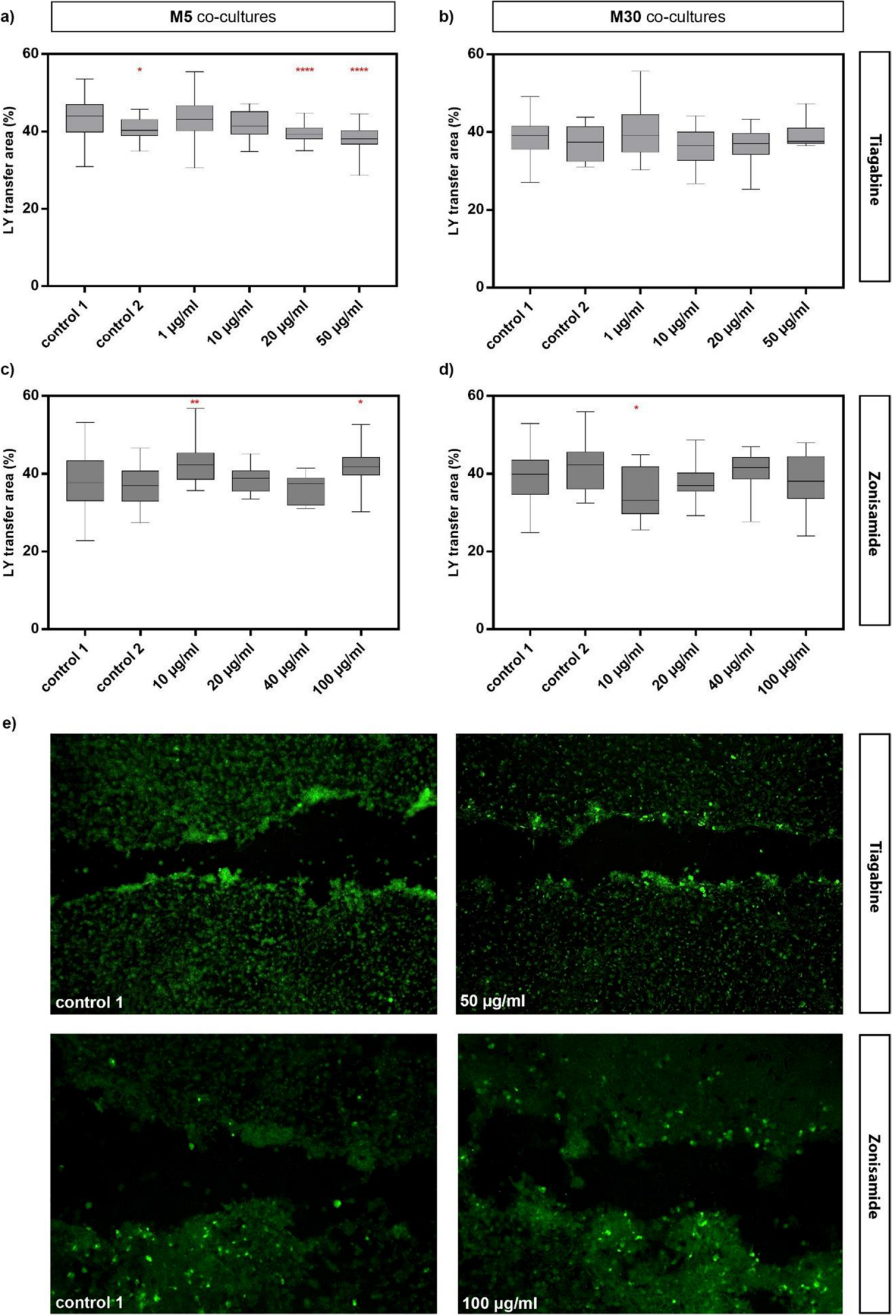
Gap-junctional cell–cell communication detected by the percentage of cells stained with Lucifer Yellow (LY), corresponding to a distribution of LY in the entire area in %, using the scrape loading method in physiological M5 and pathological M30 astrocyte-microglia co-cultures after the incubation with different concentrations of zonisamide (ZNS) (10, 20, 40 or 100 µg/ml) (**c, d**) or tiagabine (TGB) (1, 10, 20 or 50 µg/ml) (**a, b**) for 24 h. The incubation with 20 µg/ml (p < 0.0001: ****) (n = 77) and 50 µg/ml (p < 0.0001: ****) (n = 54) TGB in M5 co-cultures significantly reduced the cell–cell communication compared to control 1 (untreated cells) (**a**). Interestingly, control 2 (cells incubated with distilled H_2_O) led to a slightly significant (p < 0.05: *) (n = 54) reduction of LY-stained cells compared to control 1. The incubation with 10 and 100 µg/ml ZNS in M5 co-cultures significantly increased the LY-stained cells (p < 0.05: *; p < 0.01: **) (n = 32, n = 22, respectively) compared to both controls (**c**). The incubation with different concentrations of TGB in M30 co-cultures caused no significant changes in cell–cell communication (**b**), but incubation with 10 µg/ml ZNS resulted in a significant decrease of LY-stained cells compared to both controls (n = 22) (**d**). The panel **e** shows examples of fluorescence micrographs (LY-marked cells) of control 1 compared to the incubation with 50 μg/ml TGB, and control 1 compared to the incubation with 100 μg/ml ZNS of the physiological M5 co-culture. Comparisons between the groups were performed using the D’Agostino-Pearson normality test and one-way ANOVA followed by a Kruskal–Wallis test. Differences were considered significant at p < 0.05: *, p < 0.01: **, p < 0.0001: ****. Control 1: untreated cells with any substance/vehicle; Control 2: cells incubated with the vehicle distilled H_2_O (5 or 20 µl per ml cell culture medium)

Physiological M5 and pathological M30 co-cultures were incubated with 1, 10, 20 and 50 µg/ml TGB or 10, 20, 40 and 100 µg/ml ZNS for 24 h and the gap-junctional cell–cell communication was detected by the percentage of cells stained with LY, corresponding to a distribution of LY in the entire area in %. Cell–cell communication was significantly reduced after the incubation of M5 co-cultures with 20 and 50 µg/ml TGB compared to control 1 (untreated cells). Interestingly, control 2 (cells incubated with distilled H_2_O) revealed a slight significant reduction of LY-stained cells compared to control 1 (Fig. 5 a, e). After the incubation of M30 co-cultures with different concentrations of TGB, no significant changes in cell–cell communication were found (Fig. 5 b).

The incubation of M5 co-cultures with 10 and 100 µg/ml ZNS led to a significant increase of LY-stained cells compared to both controls (Fig. 5 c, e). A significant decrease of LY-stained cells was found after incubation with 10 µg/ml ZNS of M30 co-cultures compared to both controls (Fig. 5 d).

**Fig. 6:**
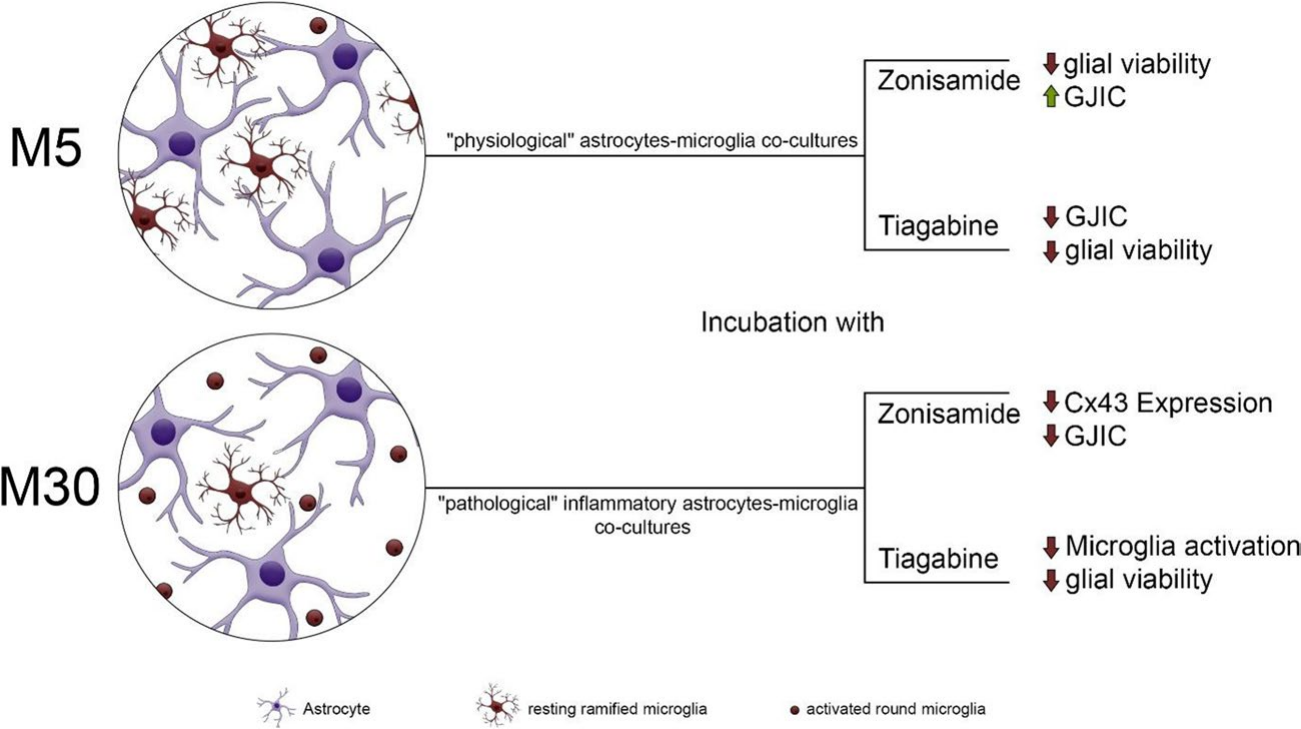
A summary of the most important findings after the incubation of astrocyte-microglia co-cultures (M5/M30) with zonisamide (ZNS) or tiagabine (TGB). The ZNS reduced the glial viability only by a high concentration under physiological conditions. By contrast, TGB led to toxic effects with a significant, concentration-dependent reduction of glial viability under physiological and pathological conditions. Under inflammatory conditions, TGB decreased the microglial activation significantly, suggesting possible anti-inflammatory features. The ZNS caused no significant changes of microglial phenotypes. The gap-junctional intercellular communication (GJIC) was significantly decreased after the incubation of M5 co-cultures with TGB, which can be related to its anti-epileptic activity under noninflammatory conditions. A significant decrease of connexin 43 (Cx43) expression and GJIC was detected after the incubation of M30 co-cultures with ZNS, suggesting additional anti-seizure effects of ZNS under inflammatory conditions

Figure 6 shows a summary of the most important findings after the incubation of astrocyte-microglia co-cultures with TGB or ZNS.

## Discussion

In this study, TGB and ZNS differentially regulated the glial properties in the astrocyte-microglia co-culture model of inflammation.

It is well-known that TGB acts as a potent inhibitor of γ-aminobutyric acid uptake in neuronal and glial cells, including astrocytes (Ängehagen et al. 2003; Brodie 1995; Fraser et al. 1999a; Leach et al. 1996; Suzdak and Jansen 1995). However, only a few studies are available regarding the additional effects of TGB on astrocytes and microglia. A previous study showed that high concentrations of TGB (50 and 100 µg/ml) significantly reduced the glial viability and increased the lactic dehydrogenase-release in primary cultures of rat astrocytes using MTT assay and spectrophotometry for lactic dehydrogenase-release analyses in the culture medium (Pavone and Cardile 2003). By contrast, treatment with TGB at low doses (1 and 10 µg/ml) did not affect the metabolic activities or exhibit cytotoxic effects on astrocytes (Pavone and Cardile 2003). Therapeutic TGB concentrations (1 and 10 µg/ml based on in vitro study findings) did not change the metabolic activities of astrocytes significantly or cause DNA damage (Cardile et al. 2000). Only high concentrations of TGB (20 and 50 µg/ml) led to DNA damage in rat astrocytes in vitro (Cardile et al. 2001). Our findings confirm previous data, showing reduced glial cell viability after a concentration-dependent incubation of physiological M5 and pathological M30 co-cultures with 10, 20 and 50 µg/ml TGB.

The TGB inhibited microglial activation and revealed neuroprotective effects in a previous study using a mouse model of Parkinson’s disease (Liu et al. 2015). Further studies aiming at pro- or anti-inflammatory features of TGB are missing. The role of microglia and inflammation in epilepto- and ictogenesis has been demonstrated previously (Devinsky et al. 2013; Eyo et al. 2017; Vezzani et al. 2011, 2013; Vezzani and Granata 2005). Evidence from human and experimental studies showed microglial activation in epileptic tissue (Devinsky et al. 2013; Eyo et al. 2017). Both molecular and morphological activation of microglia have been described in epilepsy. The morphological activation includes, for example, an increase of microglial number, and shortening and thickening of their processes with an enlargement of their cell bodies (Eyo et al. 2017). The molecular activation also encompasses upregulation of the fractalkine receptor, selectively expressed on microglia in the central nervous system, and microglial cytokine expression, including transforming growth factor (TGF)-β, interleukin (IL)-1β and TNF-α, in addition to the classical activations markers, such as Iba-1, CD68 or CD11b (Eyo et al. 2017). Therefore, we studied the effects of TGB on microglia in our co-culture model. After the treatment of physiological M5 co-cultures with 50 µg/ml TGB, the amount of resting microglia was significantly decreased with a parallel slight, but not significant increase of activated microglia. After the incubation of pathological M30 co-cultures with 20 µg/ml TGB, the microglial activation was significantly decreased and, additionally, the amount of resting microglia was slightly, but not significantly increased. These findings suggest that TGB may exert anti-inflammatory features under pathological conditions. Interestingly, the opposite effect was observed in our physiological co-cultures. These novel insights are important, because microglial cells have come into focus for anti-epileptic therapy in recent years. The membrane receptor Csf1R, which is expressed by microglia, was identified as an anti-epileptic drug target (Srivastava et al. 2018). Further research in this direction is necessary in the future.

There is strong evidence that increased GJ coupling and increased Cx43 expression are associated with epileptic activity (Gajda et al. 2003; Mylvaganam et al. 2010, 2014; Szente et al. 2002). An increased Cx43 expression was detected in human tissue from patients with epilepsy (Collignon et al. 2006; Naus et al. 1991). Astroglial Cx43 expression detected by immunocytochemistry was not affected after a concentration-dependent incubation of physiological M5 co-cultures with TGB, but a slight, significant reduction was observed after the incubation of pathological M30 co-cultures with 10 µg/ml TGB compared to controls. Because the effect was observed by only one concentration, the relevance of these minimal changes should be viewed with caution. The gap-junctional coupling was significantly reduced after the incubation of M5 co-cultures with high, overdose concentrations of TGB (20 and 50 µg/ml) compared to untreated controls, but this effect was not observed in M30 co-cultures. The reduced GJ coupling by TGB could be related to its anti-epileptic activity. Interestingly, this was the case under noninflammatory conditions but not under inflammatory conditions.

Zonisamide, a new-generation ASM, revealed neuroprotective effects against oxidative stress in astrocytes (Asanuma et al. 2010). In another study, ZNS led to neuroprotection in two animal models of Parkinson’s disease, through increasing the expression of astrocyte-mediated neurotrophic and anti-oxidative factors (Choudhury et al. 2012). Studies regarding ZNS effects on glial viability have not been available until now. The high, overdose concentration (100 µg/ml) of ZNS in our astrocyte-microglia co-culture model of inflammation reduced the glial viability only under physiological conditions. The incubation of pathological M30 co-cultures with different concentrations of ZNS revealed no significant changes. The regulatory mechanisms involved in pathological co-cultures are not known. Taken together, ZNS showed no toxic effects under therapeutic concentrations in our study.

There is only a single study showing that ZNS inhibited microglial activation in the spinal cord in a mouse model of neuropathic pain and reduced amoeboid-shaped activated microglia in BV2 microglial cell lines (Koshimizu et al. 2020). Other findings regarding ZNS effects on microglia are not available. In contrast to previous data on the inhibition of microglial activation, the incubation of our physiological M5 and pathological M30 co-cultures with different concentrations of ZNS resulted in no significant changes of microglial phenotypes. The different results are perhaps due to different study designs, for example, microglia monocultures versus co-cultures. The astrocyte-microglia co-culture model provides a good basis for further investigations of cellular interactions.

Based on previous findings from epileptic animal models and human epilepsy studies, it is known that connexin-based GJs and hemichannels are related to epilepsy (Mylvaganam et al. 2010, 2014; Samoilova et al. 2003; Szente et al. 2002). However, there has only been one previous study investigating the ZNS effects on Cx43 expression. Subchronic ZNS administration decreased the Cx43 expression and acute/subchronic administration inhibited the Cx43 hemichannel activity in the astroglial plasma membrane using primary cultured astrocytes, modulating the astroglial release of L-glutamate and adenosine triphosphate (Fukuyama et al. 2020). No significant changes of Cx43 expression were detected after the incubation of physiological M5 co-cultures with different concentrations of ZNS, but a significant reduction was found after incubation of M30 co-cultures with 10, 20 and 40 µg/ml ZNS. The incubation of M5 co-cultures with 10 and 100 µg/ml ZNS led to a significant increase of gap junctional communication, but the consequence of these changes remains unclear. A significant decrease of coupled cells was found after incubation with 10 µg/ml ZNS of M30 co-cultures consistent with decreased Cx43 expression. Taken together, ZNS may exert additional anti-seizure effects with a reduction of astrocytic Cx43 expression and disruption of gap-junctional coupling under inflammatory conditions.

Evidence based on specimens from patients with pharmacoresistant temporal lobe epilepsy and epilepsy models suggests that astrocytes in hippocampal sclerotic areas show a decreased expression of inwardly rectifying K^+^ (Kir) channels, particularly Kir4.1, leading to a decrease in spatial K^+^ buffering and an increase in neuronal excitation, contributing to seizure generation and epilepsy (Gabriel et al. 1998; Kovács et al. 2012; Steinhäuser et al. 2012; Wallraff et al. 2006). Moreover, in a blood–brain barrier breakdown and albumin-induced models of epileptogenesis, a change in the astrocytic gene expression was detected, followed by a K^+^ accumulation, leading to a frequency- and N-methyl-D-aspartate -dependent synaptic facilitation with seizure-like activity (David et al. 2009). These findings highlight the role of astrocytic dysfunction early during epileptogenesis, contributing to excitatory-inhibitory imbalance, neuronal hyperexcitability and seizure generation (David et al. 2009; Friedman and Heinemann 2012; Kovács et al. 2012). Based on experimental studies, the maturation of glial and nervous tissue was discussed to be relevant in terms of epileptogenesis. In a hippocampal model of astroglial uncoupling, for example, no change in the axonal fiber volley amplitude and amplitude of K^+^ transients after high-frequency stimulation was found in slices from young rats (3–5-week-old animals) in contrast to slices from adult rats (8–10-week-old animals) accompanied by increased amplitudes, indicating that the K^+^ channel expression and Na/K-ATPase expression probably still needs to mature (Breithausen et al. 2020). Interestingly, samples of young postnatal rats (P0–P2) included in our experiments can also be talked of as having an immature status of K^+^ channels similar to samples of patients with temporal lobe epilepsy, where channels showed a decreased expression. This is underlined by our previously reported findings that, for example, the membrane resting potential of our M5 co-culture was lower than the usual membrane resting potential (median -59.00 mV, range -29.00 mV to -77.80 mV) and median -16.30 mV (range -1.80 mV to 66.10 mV) in M30 co-culture, indicating a possible immature situation of K^+^ channels and alteration of spatial K^+^ buffering (Hinkerohe et al. 2005). In summary, our co-culture model is comparable to cell lines from patients with epilepsy, and the inflammatory M30 co-culture is a particularly useful model with features of epileptogenic tissue. Following this, it is suitable for studying effects of ASMs (Corvace et al. 2022; Dambach et al. 2014; Faustmann et al. 2022; Ismail et al. 2022; Ismail et al. 2021a).

Another point that needs to be addressed is that the TGB concentrations (1, 10, 20 or 50 µg/ml) used in our study were selected according to previous in vitro study findings (Cardile et al. 2000, 2001; Pavone and Cardile 2003) and are a magnitude higher than therapeutic plasma levels in humans, which have been reported to range between 1–234 and 43–552 ng/ml depending on the clinical studies, at doses of 30 to 56 mg or ≤ 64 mg per day (Adkins and Noble 1998; Leppik et al. 1999). Accordingly, based on our results, the human concentrations are not expected to have toxic effects on glia. On the other hand, ZNS (10, 20, 40 or 100 µg/ml) was tested at clinically relevant concentrations. The therapeutic range of plasma levels in humans for ZNS has been reported to be between 10 and 40 µg/ml (Perucca and Bialer 1996; Walker and Patsalos 1995). Consequently, the effects of ZNS in our study have higher clinical relevance.

Limitations of our study include missing investigations on inflammatory cytokines, such as TNF-α, IL-1β or IL-6, after incubation with TGB or ZNS. Additional studies of further microglial activation markers and signaling pathways after treatment with both drugs are also useful. An MTT assay was performed to detect the glial cell viability after the concentration-dependent incubation with the drugs. However, an additional cell membrane integrity assay for estimating the number of nonviable cells may be useful to provide additional evidence of cytotoxicity. Moreover, the phosphorylation status of Cx43, which is involved in the regulation of Cx43 structure and function, was not identified. In this study, we analyzed the immunocytochemical Cx43 signal per cell using the ImageJ software program. In previous studies, different methods of Cx43 evaluation were applied, such as western blot analysis or visual detection of Cx43 positivity by immunofluorescence microscopy using a rank scale in %, leading to partly divergent results (Faustmann et al. 2003; Ismail et al. 2021b). Future studies confirming the results in animal models are necessary. In addition, the gap-junctional cell–cell communication in this study was detected by the percentage of LY staining using the scrape loading method. The LY is applied as a marker for intercellular communication activity through GJs by a scrape-loading assay (including Cx43 and Cx30), but also for the establishment of proper tight junctions and neuronal morphology. Additional studies with other approaches to study the functional coupling of astrocytes are necessary to deepen our knowledge in this research field. Many different techniques are available, including recording the astrocytes membrane potential, electrophysiological methods, such as patch-clamp (commonly used for detection of gap-junctional cell–cell coupling), fluorophores, for example, Alexa fluor dyes for tracing coupled cells, genetic approaches, such as the gap-FRAP technique (fluorescence recovery after photobleaching), and techniques for the measurement of Ca^2+^ fluctuations and Ca^2+^ waves (Dong et al. 2018; Khakh and McCarthy 2015; Stephan et al. 2021).

## Conclusions

We have selected TGB and ZNS to study in our astrocyte-microglia co-culture model because of the increasing evidence confirming the involvement of astrocytes and microglia in epilepsy and limited studies of ASM effects on glial cells. Tiagabine revealed toxic effects with a concentration-dependent reduction of glial cell viability both under physiological and pathological conditions. In contrast to TGB, glial cell viability was affected by ZNS only by an overdose concentration (100 µg/ml) under physiological conditions. Zonisamide did not show any toxic effects under therapeutic concentrations in our study. After the incubation of pathological M30 co-cultures with high concentration of TGB, the microglial activation was significantly decreased and, additionally, the amount of resting microglia was slightly, but not significantly increased. These findings suggest that TGB may exert anti-inflammatory features under pathological conditions. Interestingly, the opposite effect was observed in our physiological co-cultures, with a decrease of resting microglia after treatment with increasing concentrations of TGB. Otherwise, the incubation of our physiological M5 and pathological M30 co-cultures with different concentrations of ZNS did not result in any significant changes of microglial phenotypes. The gap-junctional coupling was significantly decreased after the incubation of M5 co-cultures with high, overdose concentrations of TGB (20 and 50 µg/ml) compared to untreated controls, but this effect was not observed in M30 co-cultures. The reduced GJ coupling by TGB could be related to its anti-epileptic activity under noninflammatory conditions. A significant decrease of coupled cells was found after the incubation of M30 co-cultures with 10 µg/ml ZNS, consistent with decreased Cx43 expression. Taken together, ZNS may exert additional anti-seizure effects with a reduction of astrocytic Cx43 expression and disruption of glial gap-junctional coupling under inflammatory conditions.

Developing novel ASMs targeting glial cells, such as astrocytes and microglia, can have future potential as “add-on” therapy in addition to classical ASMs targeting neuronal cells.

## Data Availability

The data that support the findings of this study are available from the corresponding author [FSI], upon reasonable request.

## Code availability

The data that support the findings of this study are available from the corresponding author [FSI], upon reasonable request.

## References

[CR1] Adkins JC, Noble S (1998). Tiagabine. A Review of Its Pharmacodynamic and Pharmacokinetic Properties and Therapeutic Potential in the Management of Epilepsy. Drugs.

[CR2] Ängehagen M, Ben-Menachem E, Rönnbäck L, Hansson E (2003). Novel Mechanisms of Action of Three Antiepileptic Drugs, Vigabatrin, Tiagabine, and Topiramate. Neurochem Res.

[CR3] Aronica El, Bauer S, Bozzi Y, Caleo M, Dingledine R, Gorter JA, Henshall DC, Kaufer D, Koh S, Löscher W, Louboutin JP, Mishto M, Norwood BA, Palma E, Poulter MO, Terrone G, Vezzani A, Kaminski RM (2017). Neuroinflammatory Targets and Treatments for Epilepsy Validated in Experimental Models. Epilepsia.

[CR4] Asanuma M, Miyazaki I, Diaz-Corrales FJ, Kimoto N, Kikkawa Y, Takeshima M, Miyoshi Ko, Murata M (2010). Neuroprotective Effects of Zonisamide Target Astrocyte. Ann Neurol.

[CR5] Biton V (2007). Clinical Pharmacology and Mechanism of Action of Zonisamide. Clin Neuropharmacol.

[CR6] Breithausen B, Kautzmann S, Boehlen A, Steinhäuser C, Henneberger C (2020). Limited Contribution of Astroglial Gap Junction Coupling to Buffering of Extracellular K+ in CA1 Stratum Radiatum. Glia.

[CR7] Brodie MJ (1995). Tiagabine Pharmacology in Profile. Epilepsia.

[CR8] Cardile V, Pavone A, Renis M, Russo A, Perciavalle V (2000). Biological Effects of Tiagabine on Primary Cortical Astrocyte Cultures of Rat. Neurosci Lett.

[CR9] Cardile V, Palumbo M, Renis M, Pavone A, Maci T, Perciavalle V (2001). Tiagabine Treatment and DNA Damage in Rat Astrocytes: An in Vitro Study by Comet Assay. Neurosci Lett.

[CR10] Choudhury Me, Sugimoto K, Kubo M, Iwaki H, Tsujii T, Wt Kyaw N, Nishikawa M, Nagai JT, Nomoto M (2012). Zonisamide Up-Regulated the MRNAs Encoding Astrocytic Anti-Oxidative and Neurotrophic Factors. Eur J Pharmacol.

[CR11] Collignon F, Wetjen NM, Cohen-Gadol AA, Cascino GD, Parisi J, Meyer FB, Richard Marsh W, Roche P, Weigand SD (2006). Altered Expression of Connexin Subtypes in Mesial Temporal Lobe Epilepsy in Humans. J Neurosurg.

[CR12] Corvace F, Faustmann TJ, Faustmann PM, Ismail FS (2022)Anti-Inflammatory Properties of Lacosamide in an Astrocyte-Microglia Co-Culture Model of Inflammation. Eur J Pharmacol 915. 10.1016/J.EJPHAR.2021.174696 10.1016/j.ejphar.2021.174696 34902360

[CR13] Corvace F, Faustmann TJ, Heckers S, Faustmann PM, Ismail FS (2023) Experimental Investigations of Monomethyl and Dimethyl Fumarate in an Astrocyte-Microglia Co-Culture Model of Inflammation. Pharmacology 1–11. 10.1159/000528938 10.1159/000528938 36724743

[CR14] Dalmau J, Graus F (2018). Antibody-Mediated Encephalitis. N Engl J Med.

[CR15] Dambach H, Hinkerohe D, Prochnow N, Stienen MN, Moinfar Z, Haase CG, Hufnagel A, Faustmann PM (2014). Glia and Epilepsy: Experimental Investigation of Antiepileptic Drugs in an Astroglia/Microglia Co-Culture Model of Inflammation. Epilepsia.

[CR16] David Y, Cacheaux LP, Ivens S, Lapilover E, Heinemann U, Kaufer D, Friedman A (2009). Astrocytic Dysfunction in Epileptogenesis: Consequence of Altered Potassium and Glutamate Homeostasis?. J Neurosci.

[CR17] Dermietzel R, Hertzberg EL, Kessler JA, Spray DC (1991). Gap Junctions between Cultured Astrocytes: Immunocytochemical, Molecular, and Electrophysiological Analysis. J Neurosci.

[CR18] Devinsky O, Vezzani A, Najjar S, De Lanerolle NC, Rogawski MA (2013). Glia and Epilepsy: Excitability and Inflammation. Trends Neurosci.

[CR19] Dong A, Liu S, Li Y (2018) Gap Junctions in the Nervous System: Probing Functional Connections Using New Imaging Approaches. Front Cell Neurosci 12. 10.3389/FNCEL.2018.00320 10.3389/fncel.2018.00320 PMC6156252 30283305

[CR20] El-Fouly MH, Trosko JE, Chang CC (1987). Scrape-Loading and Dye Transfer. A Rapid and Simple Technique to Study Gap Junctional Intercellular Communication. Exp Cell Res.

[CR21] Eyo UB, Murugan M, Long Jun Wu (2017). Microglia-Neuron Communication in Epilepsy. Glia.

[CR22] Faustmann PM, Haase CG, Romberg S, Hinkerohe D, Szlachta D, Smikalla D, Krause D, Dermietzel R (2003). Microglia Activation Influences Dye Coupling and Cx43 Expression of the Astrocytic Network. Glia.

[CR23] Faustmann TJ, Corvace F, Faustmann PM, Ismail FS (2022). Effects of Lamotrigine and Topiramate on Glial Properties in an Astrocyte-Microglia Co-Culture Model of Inflammation. Int J Neuropsychopharmacol.

[CR24] Fiest KM, Sauro KM, Wiebe S, Patten SB, Kwon CS, Dykeman J, Pringsheim T, Lorenzetti DL, Jetté N (2017). Prevalence and Incidence of Epilepsy: A Systematic Review and Meta-Analysis of International Studies. Neurology.

[CR25] Fisher RS, Acevedo C, Arzimanoglou A, Alicia Bogacz J, Cross H, Elger CE, Engel J, Forsgren L, French JA, Glynn M, Hesdorffer DC, Lee BI, Mathern GW, Moshé SL, Perucca E, Scheffer IE, Tomson T, Watanabe M, Wiebe S (2014). ILAE Official Report: A Practical Clinical Definition of Epilepsy. Epilepsia.

[CR26] Fraser CM, Sills GJ, Butler E, Thompson GG, Lindsay K, Duncan R, Howatson A, Brodie MJ (1999). Effects of Valproate, Vigabatrin and Tiagabine on GABA Uptake into Human Astrocytes Cultured from Foetal and Adult Brain Tissue. Epileptic Disord.

[CR27] Fraser CM, Sills GJ, Forrest G, Thompson GG, Brodie MJ (1999). Effects of Anti-Epileptic Drugs on Glutamine Synthetase Activity in Mouse Brain. Br J Pharmacol.

[CR28] Friedman A and Heinemann U (2012) Role of Blood-Brain Barrier Dysfunction in Epileptogenesis. Jasper’s Basic Mech Epilepsies 353–61. 10.1093/med/9780199746545.003.0027

[CR29] Fukuyama K, Ueda Y, Okada M (2020). Effects of Carbamazepine, Lacosamide and Zonisamide on Gliotransmitter Release Associated with Activated Astroglial Hemichannels. Pharmaceuticals (basel, Switzerland).

[CR30] Gabriel S, Eilers A, Kivi A, Kovacs R, Schulze K, Lehmann TN, Heinemann U (1998). Effects of Barium on Stimulus Induced Changes in Extracellular Potassium Concentration in Area CA1 of Hippocampal Slices from Normal and Pilocarpine-Treated Epileptic Rats. Neurosci Lett.

[CR31] Gajda Z, Gyengési E, Edit Hermesz K, Ali S, Szente M (2003). Involvement of Gap Junctions in the Manifestation and Control of the Duration of Seizures in Rats in Vivo. Epilepsia.

[CR32] Geis C, Planagumà J, Carreño M, Graus F, Dalmau J (2019). Autoimmune Seizures and Epilepsy. J Clin Investig.

[CR33] Giaume C, Fromaget C, El Aoumari A, Cordier J, Glowinski J, Grost D (1991). Gap Junctions in Cultured Astrocytes: Single-Channel Currents and Characterization of Channel-Forming Protein. Neuron.

[CR34] Giaume C, Koulakoff A, Roux L, Holcman D, Rouach N (2010). Astroglial Networks: A Step Further in Neuroglial and Gliovascular Interactions. Nat Rev Neurosci.

[CR35] Haghikia A, Ladage K, Hinkerohe D, Vollmar P, Heupel K, Dermietzel R, Faustmann PM (2008). Implications of Antiinflammatory Properties of the Anticonvulsant Drug Levetiracetam in Astrocytes. J Neurosci Res.

[CR36] Hinkerohe D, Smikalla D, Haghikia A, Heupel K, Haase CG, Dermietzel R, Faustmann PM (2005). Effects of Cytokines on Microglial Phenotypes and Astroglial Coupling in an Inflammatory Coculture Model. Glia.

[CR37] Hinkerohe D, Smikalla D, Schoebel A, Haghikia A, Zoidl G, Haase CG, Schlegel U, Faustmann PM (2010). Dexamethasone Prevents LPS-Induced Microglial Activation and Astroglial Impairment in an Experimental Bacterial Meningitis Co-Culture Model. Brain Res.

[CR38] Ismail FS, Corvace F, Faustmann PM, Faustmann TJ (2021a) Pharmacological Investigations in Glia Culture Model of Inflammation. Front Cell Neurosci 15. 10.3389/FNCEL.2021.805755 10.3389/fncel.2021.805755 PMC8716582 34975415

[CR39] Ismail FS, Faustmann TJ, Corvace F, Tsvetanova A, Moinfar Z, Faustmann PM (2021b). Ammonia Induced Microglia Activation Was Associated with Limited Effects on Connexin 43 and Aquaporin 4 Expression in an Astrocyte-Microglia Co-Culture Model. BMC Neuroscience 22(1). 10.1186/S12868-021-00628-1 10.1186/s12868-021-00628-1 PMC7993489 33765917

[CR40] Ismail FS, Faustmann PM, Kümmel M-L, Förster E, Faustmann TJ, Corvace F (2022) Brivaracetam Exhibits Mild Pro-Inflammatory Features in an in Vitro Astrocyte-Microglia Co-Culture Model of Inflammation. Front Cell Neurosci 16. 10.3389/FNCEL.2022.995861 10.3389/fncel.2022.995861 PMC9670320 36406753

[CR41] Ismail FS, Faustmann PM (2021). Experimental Investigations of Antiepileptic Drugs in Astrocytes-Microglia Co-Cultures Suggest Possible Protective Effects on Astrocytes during Early Epileptogenesis. Epilepsia.

[CR42] Khakh BS, McCarthy KD (2015)Astrocyte Calcium Signaling: From Observations to Functions and the Challenges Therein. Cold Spring Harb Perspect Biol 7(4). 10.1101/CSHPERSPECT.A020404 10.1101/cshperspect.a020404 PMC4382738 25605709

[CR43] Koshimizu H, Ohkawara B, Nakashima H, Ota K, Kanbara S, Inoue T, Tomita H, Sayo A, Kiryu-Seo S, Konishi H, Ito M, Masuda A, Ishiguro N, Imagama S, Kiyama H, Ohno K (2020) Zonisamide Ameliorates Neuropathic Pain Partly by Suppressing Microglial Activation in the Spinal Cord in a Mouse Model. Life Sci 263. 10.1016/J.LFS.2020.118577 10.1016/j.lfs.2020.118577 33058918

[CR44] Kovács R, Heinemann U, Steinhäuser C (2012). Mechanisms Underlying Blood-Brain Barrier Dysfunction in Brain Pathology and Epileptogenesis: Role of Astroglia. Epilepsia.

[CR45] Leach JP, Sills GJ, Majid A, Butler E, Carswell A, Thompson GG, Brodie MJ (1996). Effects of Tiagabine and Vigabatrin on GABA Uptake into Primary Cultures of Rat Cortical Astrocytes. Seizure.

[CR46] Leppik IE, Gram L, Deaton R, Sommerville KW (1999). Safety of Tiagabine: Summary of 53 Trials. Epilepsy Res.

[CR47] Liu J, Huang D, Xu J, Tong J, Wang Z, Huang L, Yang Y, Bai X, Wang P, Suo H, Ma Y, Yu M, Fei J, Huang F (2015) Tiagabine Protects Dopaminergic Neurons against Neurotoxins by Inhibiting Microglial Activation. Sci Rep 5. 10.1038/SREP15720 10.1038/srep15720 PMC4620555 26499517

[CR48] Löscher W, Potschka H, Sisodiya SM, Vezzani A (2020). Drug Resistance in Epilepsy: Clinical Impact, Potential Mechanisms, and New Innovative Treatment Options. Pharmacol Rev.

[CR49] Masuda Y, Utsui Y, Shiraishi Y, Karasawa T, Yoshida K, Shimizu M (1979). Relationships between Plasma Concentrations of Diphenylhydantoin, Phenobarbital, Carbamazepine, and 3-Sulfamoylmethyl-1,2-Benzisoxazole (AD-810), a New Anticonvulsant Agent, and Their Anticonvulsant or Neurotoxic Effects in Experimental Animals. Epilepsia.

[CR50] McCormick DA, Contreras D (2001). On the Cellular and Network Bases of Epileptic Seizures. Annu Rev Physiol.

[CR51] Mylvaganam S, Zhang L, Chiping Wu, Zhang ZJ, Samoilova M, Eubanks J, Carlen PL, Poulter MO (2010). Hippocampal Seizures Alter the Expression of the Pannexin and Connexin Transcriptome. J Neurochem.

[CR52] Mylvaganam S, Ramani M, Krawczyk M, Carlen PL (2014) Roles of Gap Junctions, Connexins, and Pannexins in Epilepsy. Front Physiol 5. 10.3389/FPHYS.2014.00172 10.3389/fphys.2014.00172 PMC4019879 24847276

[CR53] Naus CCG, Bechberger JF, Paul DL (1991). Gap Junction Gene Expression in Human Seizure Disorder. Exp Neurol.

[CR54] Ngugi AK, Kariuki SM, Bottomley C, Kleinschmidt I, Sander JW, Newton CR (2011). Incidence of Epilepsy: A Systematic Review and Meta-Analysis. Neurology.

[CR55] Pascual O, Achour SB, Rostaing P, Triller A, Bessis A (2012) Microglia Activation Triggers Astrocyte-Mediated Modulation of Excitatory Neurotransmission. Proc Natl Acad Sci U S A 109(4). 10.1073/PNAS.1111098109 10.1073/pnas.1111098109 PMC3268269 22167804

[CR56] Patel DC, Tewari BP, Chaunsali L, Sontheimer H (2019). Neuron-Glia Interactions in the Pathophysiology of Epilepsy. Nat Rev Neurosci.

[CR57] Pavone A, Cardile V (2003). An in Vitro Study of New Antiepileptic Drugs and Astrocytes. Epilepsia.

[CR58] Perucca E (1999) The Clinical Pharmacokinetics of the New Antiepileptic Drugs. Epilepsia 40 Suppl 9(SUPPL. 9). 10.1111/J.1528-1157.1999.TB02088.X 10.1111/j.1528-1157.1999.tb02088.x 10612356

[CR59] Perucca E, Bialer M (1996). The Clinical Pharmacokinetics of the Newer Antiepileptic Drugs. Focus on Topiramate, Zonisamide and Tiagabine. Clin Pharmacokinet.

[CR60] Perucca E, Tomson T (2011). The Pharmacological Treatment of Epilepsy in Adults. Lancet Neurol.

[CR61] Perucca E, French J, Bialer M (2007). Development of New Antiepileptic Drugs: Challenges, Incentives, and Recent Advances. Lancet Neurol.

[CR62] Rogawski MA, Löscher W, Rho JM (2016). Mechanisms of Action of Antiseizure Drugs and the Ketogenic Diet. Cold Spring Harb Perspect Med.

[CR63] Samoilova M, Li J, Pelletier MR, Wentlandt K, Adamchik Y, Naus CC, Carlen PL (2003). Epileptiform Activity in Hippocampal Slice Cultures Exposed Chronically to Bicuculline: Increased Gap Junctional Function and Expression. J Neurochem.

[CR64] Siracusa R, Fusco R, Cuzzocrea S (2019) Astrocytes: Role and Functions in Brain Pathologies. Front Pharmacol 10(SEP). 10.3389/FPHAR.2019.01114 10.3389/fphar.2019.01114 PMC6777416 31611796

[CR65] Srivastava PK, van Eyll J, Godard P, Mazzuferi M, Delahaye-Duriez A, Van Steenwinckel J, Gressens P, Danis B, Vandenplas C, Foerch P, Leclercq K, Mairet-Coello G, Cardenas A, Vanclef F, Laaniste L, Niespodziany I, Keaney J, Gasser J, Gillet G, Shkura K, Ah Chong S, Behmoaras J, Kadiu I, Petretto E, Kaminski RM, Johnson MR (2018) A Systems-Level Framework for Drug Discovery Identifies Csf1R as an Anti-Epileptic Drug Target. Nat Commun 9(1). 10.1038/S41467-018-06008-4 10.1038/s41467-018-06008-4 PMC6120885 30177815

[CR66] Steinhäuser C, Seifert G, Bedner P (2012). Astrocyte Dysfunction in Temporal Lobe Epilepsy: K+ Channels and Gap Junction Coupling. Glia.

[CR67] Stephan J, Eitelmann S, Zhou M (2021) Approaches to Study Gap Junctional Coupling. Front Cell Neurosci 15. 10.3389/FNCEL.2021.640406 10.3389/fncel.2021.640406 PMC7987795 33776652

[CR68] Stienen MN, Haghikia A, Dambach H, Thöne J, Wiemann M, Gold R, Chan A, Dermietzel R, Faustmann PM, Hinkerohe D, Prochnow N (2011). Anti-Inflammatory Effects of the Anticonvulsant Drug Levetiracetam on Electrophysiological Properties of Astroglia Are Mediated via TGFβ1 Regulation. Br J Pharmacol.

[CR69] Suzdak PD, Jansen JA (1995). A Review of the Preclinical Pharmacology of Tiagabine: A Potent and Selective Anticonvulsant GABA Uptake Inhibitor. Epilepsia.

[CR70] Szente M, Gajda Z, Said Ali K, Hermesz E (2002). Involvement of Electrical Coupling in the in Vivo Ictal Epileptiform Activity Induced by 4-Aminopyridine in the Neocortex. Neuroscience.

[CR71] Thijs RD, Surges R, O’Brien TJ, Sander JW (2019). Epilepsy in Adults. Lancet (london, England).

[CR72] Vezzani A, Granata T (2005). Vezzani, A., & Granata, T. (2005). Brain Inflammation in Epilepsy: Experimental and Clinical Evidence. Epilepsia, 46(11), 1724–1743. Https://Doi.Org/10.1111/J.1528-1167.2005.00298.XBrain Inflammation in Epilepsy: Experimental and Clinical Evidence. Epilepsia.

[CR73] Vezzani A, French J, Bartfai T, Baram TZ (2011). The Role of Inflammation in Epilepsy. Nat Rev Neurol.

[CR74] Vezzani A, Friedman A, Dingledine RJ (2013). The Role of Inflammation in Epileptogenesis. Neuropharmacology.

[CR75] Walker MC, Patsalos PN (1995). Clinical Pharmacokinetics of New Antiepileptic Drugs. Pharmacol Ther.

[CR76] Wallraff A, Köhling R, Heinemann U, Theis M, Willecke K, Steinhäuser C (2006). The Impact of Astrocytic Gap Junctional Coupling on Potassium Buffering in the Hippocampus. J Neurosci.

[CR77] Wang X, Ratnaraj N, Patsalos PN (2004). The Pharmacokinetic Inter-Relationship of Tiagabine in Blood, Cerebrospinal Fluid and Brain Extracellular Fluid (Frontal Cortex and Hippocampus). Seizure.

